# An Elemental Diet Enriched in Amino Acids Alters the Gut Microbial Community and Prevents Colonic Mucus Degradation in Mice with Colitis

**DOI:** 10.1128/msystems.00883-22

**Published:** 2022-12-05

**Authors:** Bowei Zhang, Congying Zhao, Xuejiao Zhang, Xiang Li, Yunhui Zhang, Xiaoxia Liu, Jia Yin, Xinyang Li, Jin Wang, Shuo Wang

**Affiliations:** a School of Medicine, Nankai University, Tianjin, China; Vall d’Hebron Institut de Recerca

**Keywords:** elemental diet, gut microbiota, mucin degradation, mucus layer

## Abstract

The role of dietary amino acids or intact proteins in the progression of colitis remains controversial, and the mechanism involving gut microbes is unclear. Here, we investigated the effects of an elemental diet (ED) enriched in amino acids and a polymeric diet enriched in intact protein on the pathogenesis of dextran sulfate sodium (DSS)-induced colitis in mice. Our results showed that the ED induced remission of colitis in mice. Notably, ED treatment reduced the abundance of the mucolytic bacteria *Akkermansia* and *Bacteroides*, which was attributed to decreased colonic protein fermentation. Consistently, the activities of mucolytic enzymes were decreased, leading to protection against mucus layer degradation and microbial invasion. Fecal microbiota transplantation from ED-fed mice reshaped microbial ecology and alleviated intestinal inflammation in recipient mice. The ED failed to induce remission of colitis in pseudogermfree mice. Together, our results demonstrate the critical role of the gut microbiota in the prevention of colitis by an ED.

**IMPORTANCE** The prevalence of inflammatory bowel disease is rapidly increasing and has become a global burden. Several specific amino acids have been shown to benefit mucosal healing and colitis remission. However, the role of amino acids or intact proteins in diets and enteral nutrition formulas is controversial, and the mechanisms involving gut microbes remain unclear. In this study, we investigated the effects of an elemental diet (ED) enriched in amino acids and a polymeric diet enriched in intact protein on the pathogenesis of colitis in mice. The underlying mechanisms were explored by utilizing fecal microbiota transplantation and pseudogermfree mice. ED treatment reduced the abundance of mucolytic bacteria, thereby protecting the mucus layer from microbial invasion and degradation. For the first time, we convincingly demonstrated the critical role of gut microbiota in the effects of the ED. This study may provide new insights into the gut microbiota-diet interaction and its role in human health.

## INTRODUCTION

Inflammatory bowel disease (IBD), including ulcerative colitis (UC) and Crohn’s disease (CD), is characterized by abdominal pain, diarrhea, and pus and blood in the stool (1). The prevalence of IBD is highest in Europe and North America, with a rapidly increasing trend in developing countries. The disease is gradually being found among younger people (2), and it has become a global burden (3).

The pathological changes of IBD mainly occur in the colonic mucosa and submucosa and gradually spread to the entire colon (4). Disruption of the intestinal flora and damage of the intestinal mucosa are the main features of IBD and are important factors in the aggravation of intestinal inflammation (5, 6). The mucus layer of the colon and intestine is the first barrier that protects the gut from bacteria (7). The colonic mucus layer is divided into an outer layer and an inner layer, the trunk of which is mainly composed of Muc2 secreted by goblet cells (8, 9). A key nutritional feature of the intestinal mucus layer is the high content of polysaccharide, of which the content of O-glycan is up to 80% (10). However, only a subset of gut microbes are capable of utilizing this nutrient source (11). In a healthy state, bacteria adhere to the outer mucus layer. Some bacteria, such as Akkermansia muciniphila and *Bacteroides*, can degrade mucus and produce short-chain fatty acids, which can provide energy for goblet cells and promote mucus secretion to maintain the sterile state of the inner mucus layer (12, 13). However, in the case of colitis, goblet cells are destroyed and mucolytic bacteria proliferate. This results in a thinning of the intestinal mucus layer, allowing bacteria to invade the intestinal epithelium, leading to a more severe inflammatory response (6, 14). Currently, several treatments for colitis are available, including nontargeted therapies such as 5-aminosalicylic acid (5-ASA), glucocorticoids, and immunosuppressants (azathioprine) (15), as well as targeted biologics, including anti-tumor necrosis factor (TNF) therapy and c-Jun N-terminal kinase (JNK) inhibitors (16). However, long-term use of these drugs may cause side effects. The pathogenesis of IBD involves both genetic and environmental factors. Among them, diet plays an important role in the progression of IBD (17). Dietary intervention strategies are more acceptable to patients and have fewer side effects, and this approach has attracted widespread attention (18).

Nutrients act as critical regulators of the immune system and gut microbial ecology (19). Among nutrients, several specific dietary amino acids participate in cellular and microbial metabolic pathways and play a role in mucosal healing and gut microbiota shaping (20, 21). An amino acid-enriched diet reduces dietary antigens in the gut lumen and is generally considered to be better absorbed in the proximal small intestine, while the residual amount in the distal small intestine and colon is minimal (22). In previous studies, mice with colitis were fed an amino acid-based elemental diet (ED) and a polymeric diet with intact proteins, and it was found that the amino acid diet inhibited colon inflammation in the mice and suppressed Th1 and Th17 cell responses (23, 24). Moreover, as a formula for enteral nutrition (EN), an ED has been shown to induce remission of IBD in patients (18). At the same time, studies comparing different EN treatments have yielded conflicting results. Earlier researches suggest that CD patients treated with an ED have significantly higher remission rates than those on a polymeric diet (25). However, later studies found that the ED was as effective as polymeric diets (26). Given the critical role of gut microbes in the progression of IBD, these inconsistent results may reflect individual differences in gut microbes. An ED can alter the gut microbial community by altering the nutrient composition of the gut microbiota (18), but information on the underlying mechanisms by which the ED alleviates IBD is still lacking.

Therefore, this study aimed to investigate the effect of an ED on the progression of colitis in mice. By utilizing fecal microbiota transplantation and antibiotic-treated pseudosterile mouse models, we elucidated the molecular mechanisms by which the ED alters gut microbiomes and affects the progression of colitis.

## RESULTS

### The elemental diet prevents the progression of colitis in mice.

**FIG 1:**
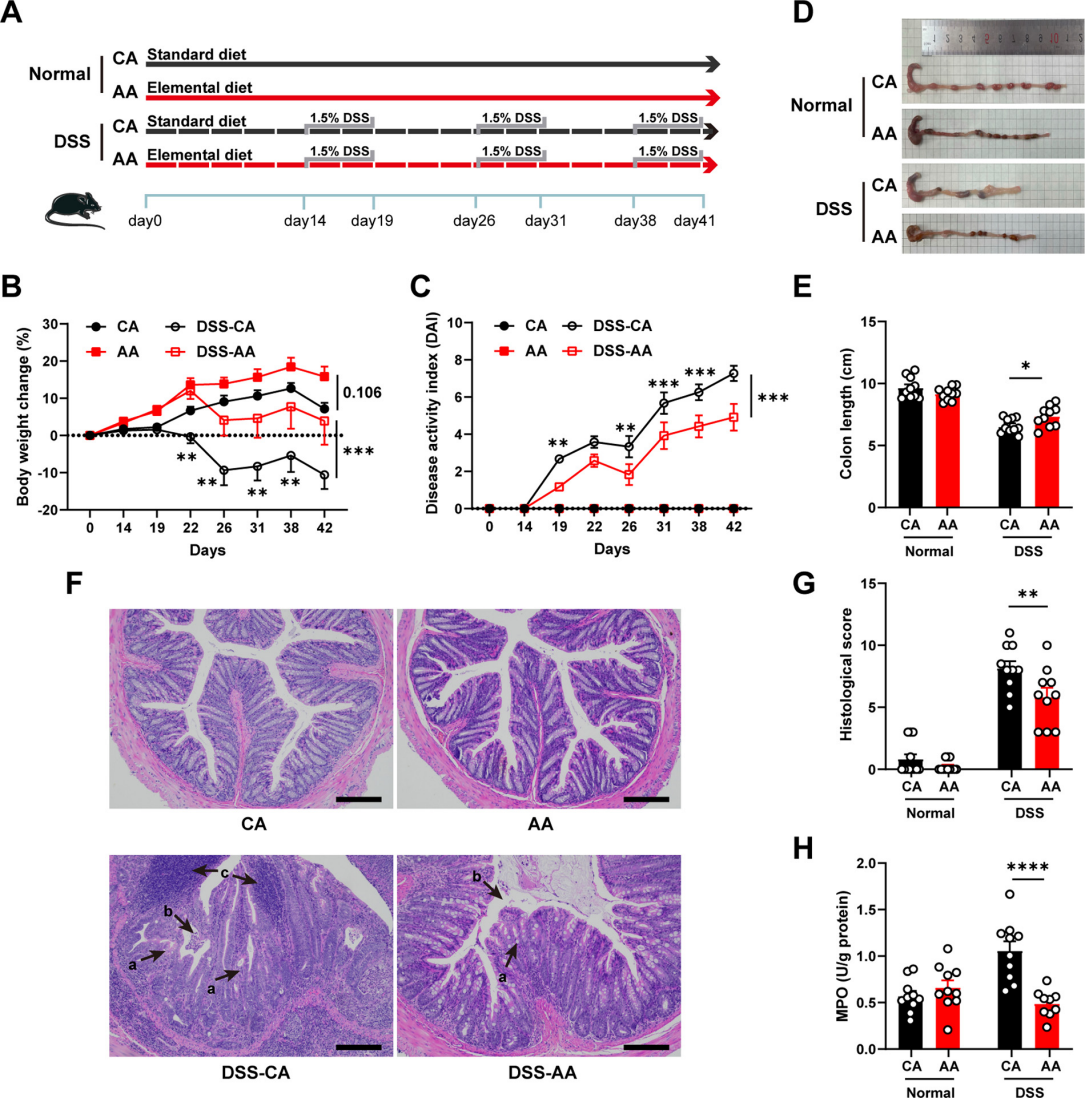
The elemental diet prevents the progression of DSS-induced colitis in mice. (A) Schematic illustration of the experimental design. (B) Body weight. (C) Disease activity index. (D) Colon images. (E) Colon length. (F) H&E-stained sections of colon tissue. Arrows show crypt deformation (a), epithelial damage (b), and infiltration of inflammatory cells in the submucosa (c). Magnification, ×100. Bar = 200 μm. (G and H) Histological scores. (H) Colonic MPO activity. Data are means and SEM, and two-way ANOVA followed by Bonferroni’s multiple-comparison test (*n* = 10) was used to determine significance. *, *P* < 0.05; **, *P* < 0.01; ***, *P* < 0.001; ****, *P* < 0.0001.

To evaluate the effect of an ED on chronic colitis, mice were fed an ED enriched in amino acids (AA) or a standard diet enriched in intact casein (CA) for 2 weeks and then given dextran sulfate sodium (DSS) for 3 cycles (Fig. 1A). During the final stage of the experiment, the disease activity index (DAI) of the DSS-treated mice gradually increased. The mice developed severe diarrhea and blood in the stool, and their body weight was significantly reduced (Fig. 1B and C). The intervention of the ED significantly alleviated these symptoms. The shortened colon is an important indicator of colitis. DSS significantly reduced the colon length in mice, but this was effectively prevented by the ED (Fig. 1D and E). Histopathology showed that mice with colitis showed crypt deformation, epithelial damage, and obvious infiltration of inflammatory cells in the submucosa, which were improved by ED treatment (Fig. 1F and G). DSS-induced colitis significantly increased the neutrophil marker myeloperoxidase (MPO), but ED treatment decreased it significantly (Fig. 1H). These data suggested that ED intervention can effectively improve the pathological damage caused by DSS-induced colitis. Interestingly, there was no significant difference in colitis-related parameters between the two diets in normal mice, but the body weight of the AA group mice was slightly increased (Fig. 1B).

### The elemental diet inhibits intestinal inflammation in mice.

**FIG 2:**
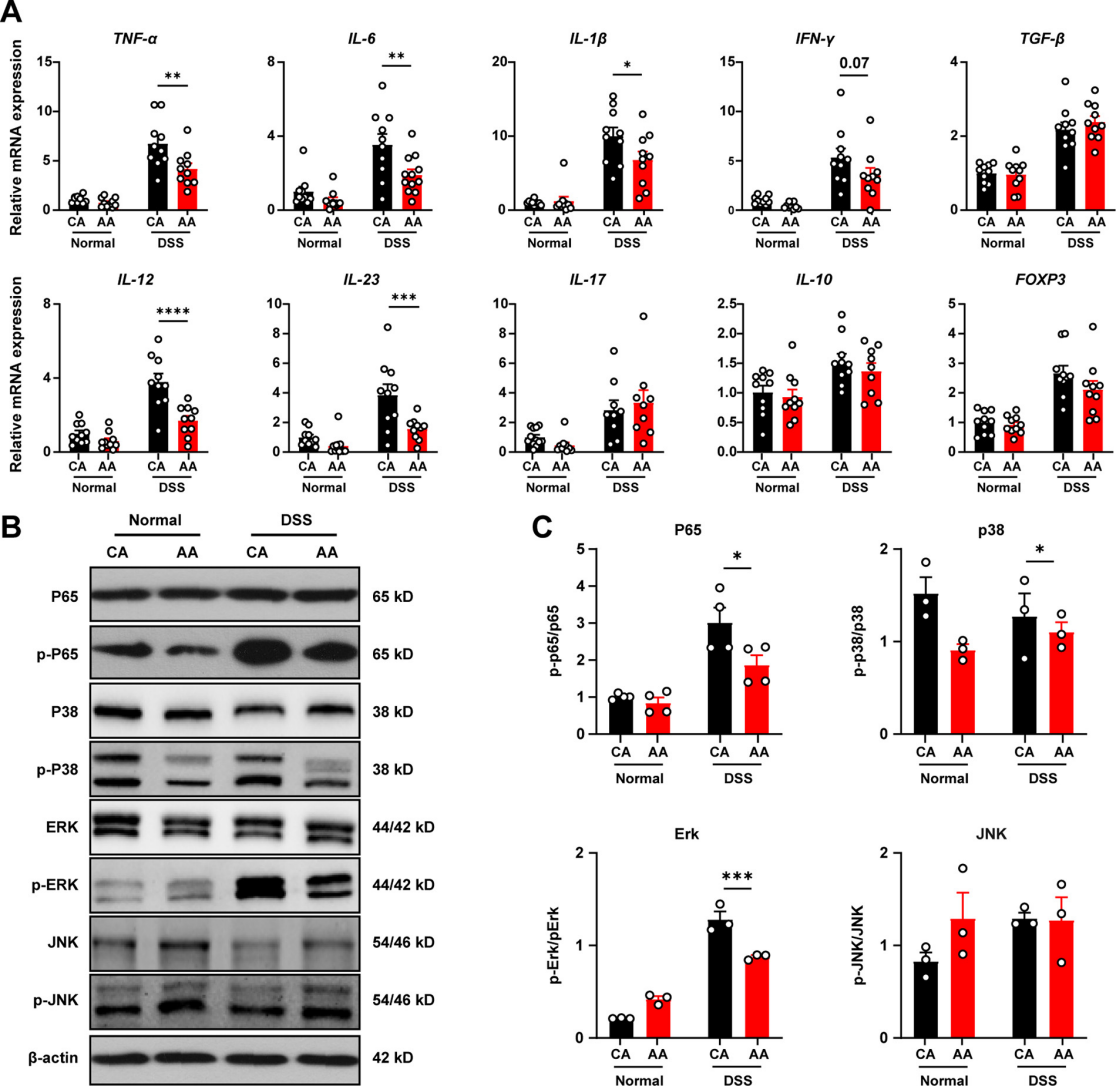
The elemental diet inhibits intestinal inflammation in mice. (A) Relative mRNA expressions of cytokines in colon tissue. (B and C) Expression of NF-κB and MAPK pathway proteins in colon tissues (B and C). Data are means and SEM, and two-way ANOVA followed by Bonferroni’s multiple-comparison test (*n* = 10 for A; *n* = 3 or 4 for B and C) was used. *, *P* < 0.05; **, *P* < 0.01; ***, *P* < 0.001; ****, *P* < 0.0001.

We then examined the mRNA expression of colitis-related proteins to explore the mechanism by which the ED alleviates colitis. The results showed that the expressions of proinflammatory factors, including interleukin 6 (IL-6), TNF-α, gamma interferon (IFN-γ), IL-12, and IL-23, was significantly downregulated in the DSS-AA group compared with the DSS-CA group (Fig. 2A). The expression of IL-1β, which is associated with inflammasome activation, was significantly decreased in the DSS-AA-treated group, but the expression of IL-17, which is associated with Th17 cell differentiation, was not significantly altered.

Western blot results showed that the phosphorylation levels of p65, Erk, and p38 in the DSS-AA group were significantly lower than those in the DSS-CA group (Fig. 2B and C). This indicates that the ED could inhibit the activation of inflammatory signaling pathways NF-κB and mitogen-activated protein kinase (MAPK), thereby reducing the release of downstream inflammatory factors and improving colitis in mice.

### Elemental diet increased mucin expression.

**FIG 3:**
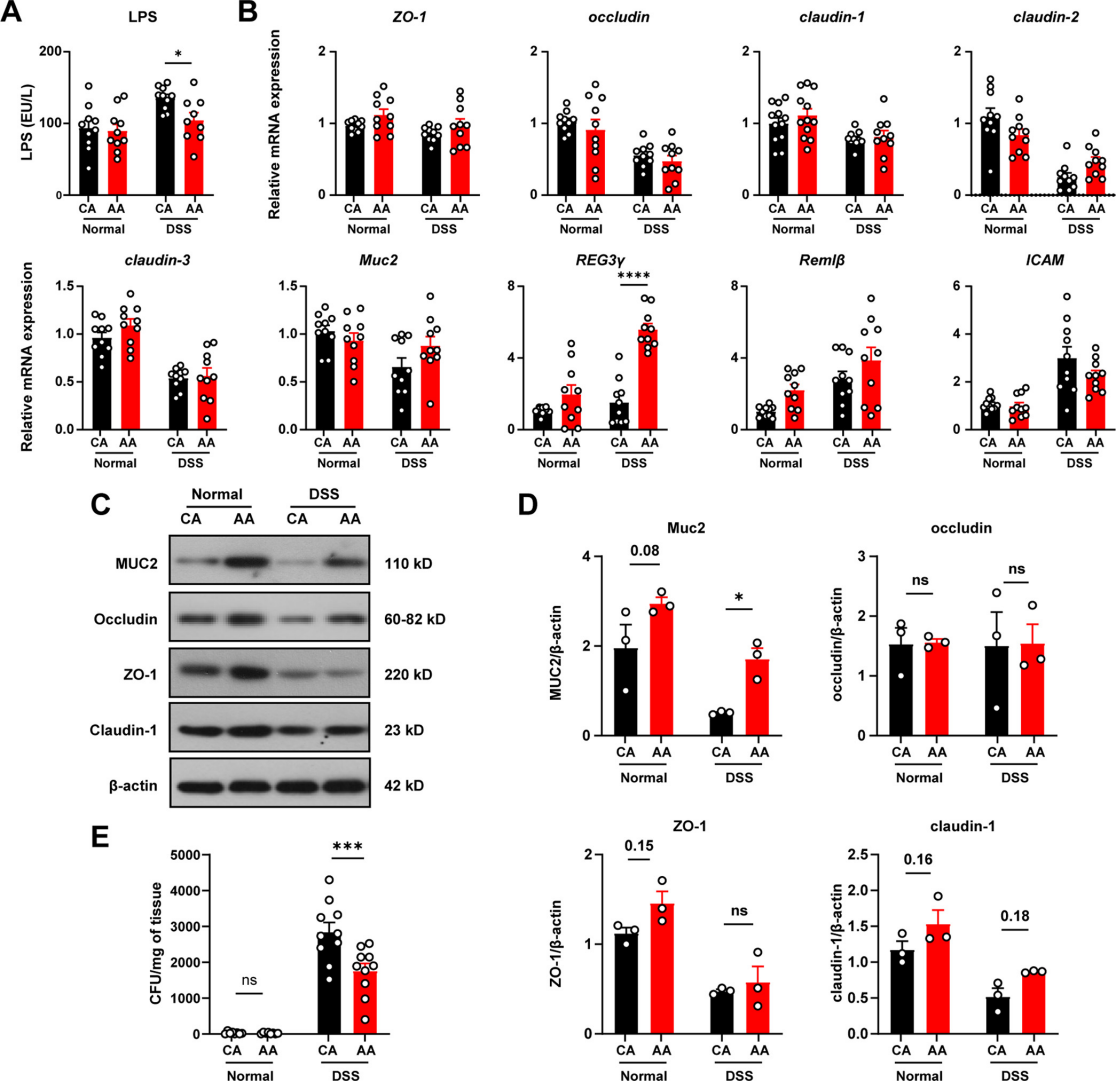
The elemental diet increases mucin expression. (A) LPS level in serum. (B) Relative mRNA expression of gut barrier-related proteins. (C and D) Expression level of gut barrier-related protein. (E) Quantification of aerobic bacteria in mesenteric lymph nodes. Data are means and SEM, and two-way ANOVA followed by Bonferroni multiple-comparison test (*n* = 10 for A, B and E; *n* = 3 for C and D) was used. *, *P* < 0.05; ***, *P* < 0.001; ****, *P* < 0.0001; ns, not significant.

Disruption of the epithelial barrier is a key driver of intestinal inflammation. We observed that the serum level of lipopolysaccharide (LPS) in the DSS-AA group was decreased compared with that in the DSS-CA group (Fig. 3A). Therefore, we further examined the mRNA expression of intestinal barrier-related proteins. In the DSS-AA group, the mRNA expression of the antibacterial peptide RegIIIγ secreted by Paneth cells was significantly increased (Fig. 3B). However, the mRNA expression of most key proteins such as Muc2, occludin, ZO-1, and claudin was comparable between the two groups.

According to the results of Western blotting, there was no significant difference in the expression of ZO-1, occludin, and claudin-1 between the DSS-AA group and the DSS-CA group. Interestingly, the amino acid-based ED increased the protein expression of Muc2 in both normal mice and those with colitis (Fig. 3C and D). Muc2 is a major component of the colonic mucus layer, which helps to resist the invasion of intestinal pathogens. Correspondingly, we observed decreased microbial translocation in the mesenteric lymph nodes of ED-fed mice (Fig. 3E). The above results indicated that the improvement of colitis by the ED was mediated by the regulation of intestinal mucins, and this did not involve changes at the transcriptional level.

### The elemental diet reduces mucolytic bacterial abundance and mucolytic enzyme activity.

**FIG 4:**
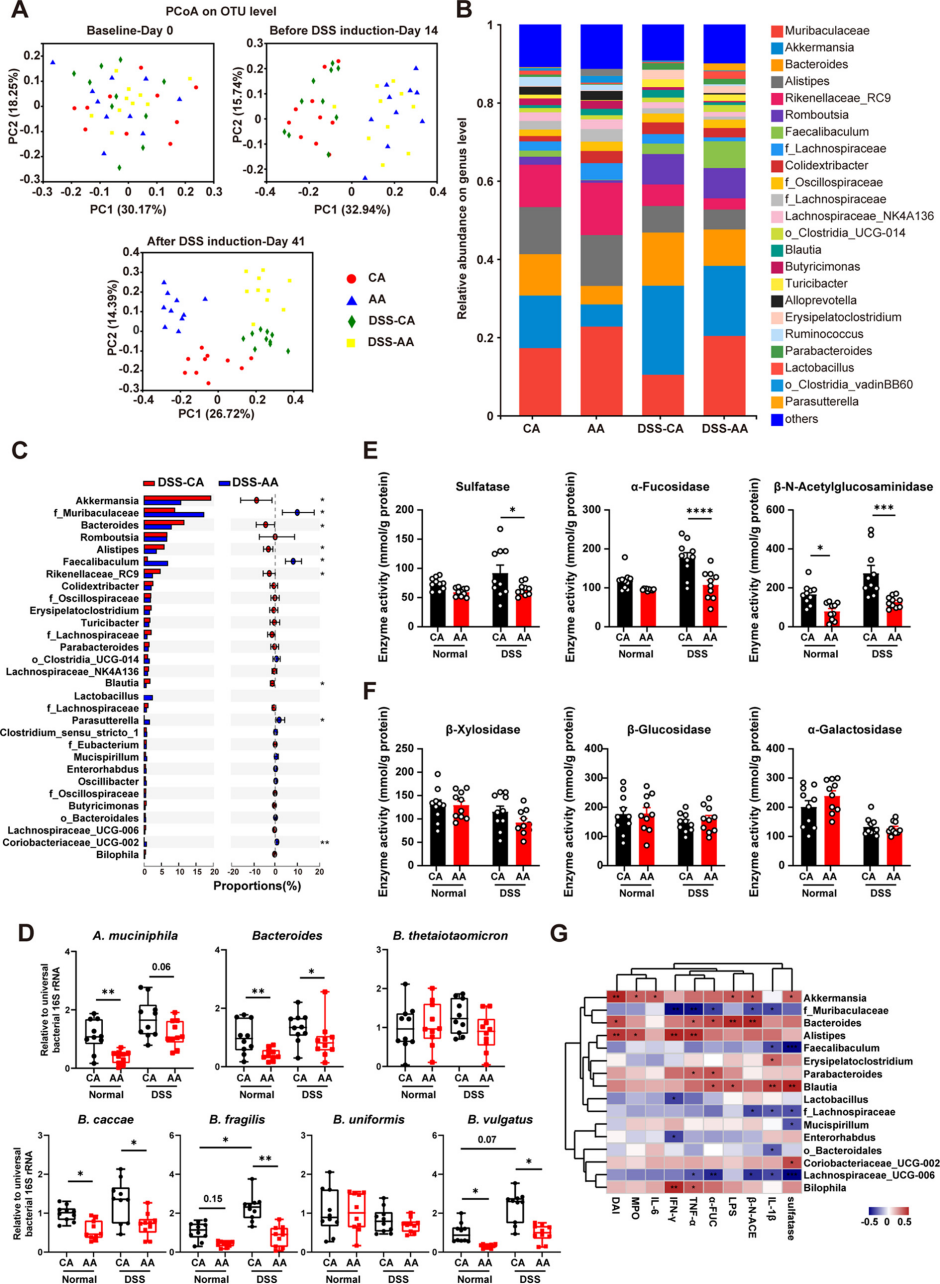
The elemental diet reduces mucolytic bacterial abundance and mucolytic enzyme activity. (A) Principal-coordinate analysis (PCoA) of fecal microbiota at the level of operational taxonomic units (OTUs). (B) Relative abundance of gut microbes at the genus level. (C) Difference between gut microbiotas in the DSS-CA group and the DSS-AA group at the genus level. (D) Ratio of relative abundance of gut microbes measured by qPCR. (E and F) Activities of mucolytic enzymes and carbohydrate-active enzymes. (G) Spearman correlation analysis. Data are means and SEM; significance was determined by the Mann-Whitney test (C) (*n* = 10), the Kruskal-Wallis test followed by Dunn’s multiple-comparison tests (D) (*n* = 10), and two-way ANOVA followed by Bonferroni’s multiple-comparison tests (E and F) (*n* = 10). *, *P* < 0.05; **, *P* < 0.01; ***, *P* < 0.001; ****, *P* < 0.0001.

To further explore the underlying mechanism, we determined the relative abundance of the gut microbiota by 16S rRNA gene sequencing. According to the results of principal-coordinate analysis (PCoA), the compositions of gut microbiotas in all groups were similar at the baseline time point (Fig. 4A; also, Fig. S1 see in the supplemental material). After 14 days of feeding, there was a significant difference in the overall composition of the gut microbiota between AA diet-fed mice and CA diet-fed mice. After DSS induction, the samples in the DSS-CA group were distinguished from those in the DSS-AA group. This difference was also observed between the CA group and the AA group, suggesting that the ED altered the composition of gut microbiota.

10.1128/msystems.00883-22.5 FIG S1 Statistical analysis of PCoA. The differences for PC1 and PC2 among groups at baseline (A) and before (B) and after (C) DSS induction are shown. Significance was examined using the Kruskal-Wallis test followed by Dunn’s multiple-comparison tests (*n* = 10). *, *P* < 0.05; **, *P* < 0.01; ***, *P* < 0.001. Download FIG S1, TIF file, 0.6 MB. Copyright © 2022 Zhang et al. 2022 Zhang et al. https://creativecommons.org/licenses/by/4.0/ This content is distributed under the terms of the Creative Commons Attribution 4.0 International license.

The ED decreased the abundance of *Akkermansia* and *Bacteroides* and increased the abundance of *Muribaculaceae* and *Faecalibaculum* in both the DSS-treated and normal groups (Fig. 4B and C and Fig. S2). The abundances of these microbes in the samples were 13.7%, 9.1%, 18.4%, and 2.5%, respectively. Furthermore, the ED decreased the abundance of *Alistipes*, *Rikenellaceae* RC9, and *Blautia* and increased the abundance of *Parasutterella* and *Coriobacteriaceae* UCG-002 in DSS-treated mice. Among the microbes with high abundance, *Bacteroide*s and *Akkermansia* were reported to be major contributors to mucus degradation (6). The abundance of *A. muciniphila* and *Bacteroides* was further determined by qPCR. The ED significantly reduced the relative abundance of *A. muciniphila* and *Bacteroides* in both normal mice and mice with colitis (Fig. 4D). Among the most abundant species of *Bacteroides* in murine gut microbiota (6, 27, 28), Bacteroides thetaiotaomicron, B. fragilis, B. caccae, B. vulgatus, B. uniformis, and B. ovatus have been reported to be mucin degraders (6, 29). According to the results of quantitative PCR (qPCR), the ED reversed the increase in the abundance of B. fragilis and B. vulgatus caused by DSS treatment and reduced the abundance of *B. caccae* (Fig. 4D). *B. ovatus* was not detected in samples in this study. The above results showed that the ED altered the gut microbial community of mice.

10.1128/msystems.00883-22.6 FIG S2 Difference between gut microbiotas in the CA group and the AA group. Data are means and SEM; significance was examined by the Mann-Whitney test (*n* = 10). *, *P* < 0.05; **, *P* < 0.01; ***, *P* < 0.001. Download FIG S2, TIF file, 0.4 MB. Copyright © 2022 Zhang et al. 2022 Zhang et al. https://creativecommons.org/licenses/by/4.0/ This content is distributed under the terms of the Creative Commons Attribution 4.0 International license.

Muc2 is an O-glycan protein linked by O-glycosidic and disulfide bonds, which can be degraded by microbial mucolytic enzymes and serve as a carbon source for microorganisms. Therefore, we measured the activity of these glycosidases in feces. In the DSS-treated mice, the activities of mucin-degrading enzymes, including sulfatase, β-*N*-acetylglucosaminidase, and α-fucosidase, were significantly increased (*P* < 0.05), and this effect was significantly reversed by the ED (Fig. 4E). Moreover, the activities of α-galactosidase, β-xylosidase, and β-glucosidase, which are involved in the degradation of plant carbohydrates, were comparable between the CA and the AA groups (Fig. 4F).

To verify the role of specific gut microbes in colitis, we performed Spearman correlation analysis between the gut microbiota and key host indicators. The results showed that the abundance of the mucolytic bacteria *Bacteroide*s and *Akkermansia* was positively correlated with mucolytic enzyme activities, DAI, LPS levels, and inflammatory-cytokine levels (Fig. 4G), and the same was true for *Alistipes* and *Blautia*. The abundance of *Muribaculaceae* and *Lachnospiraceae* UCG-006 was negatively correlated with many of the indicators.

In brief, these results suggest that changes in the microbial community, especially mucus-degrading bacteria, may play an important role in the prevention of colitis by the ED.

### Prevention of colitis by the elemental diet is microbiota dependent.

**FIG 5:**
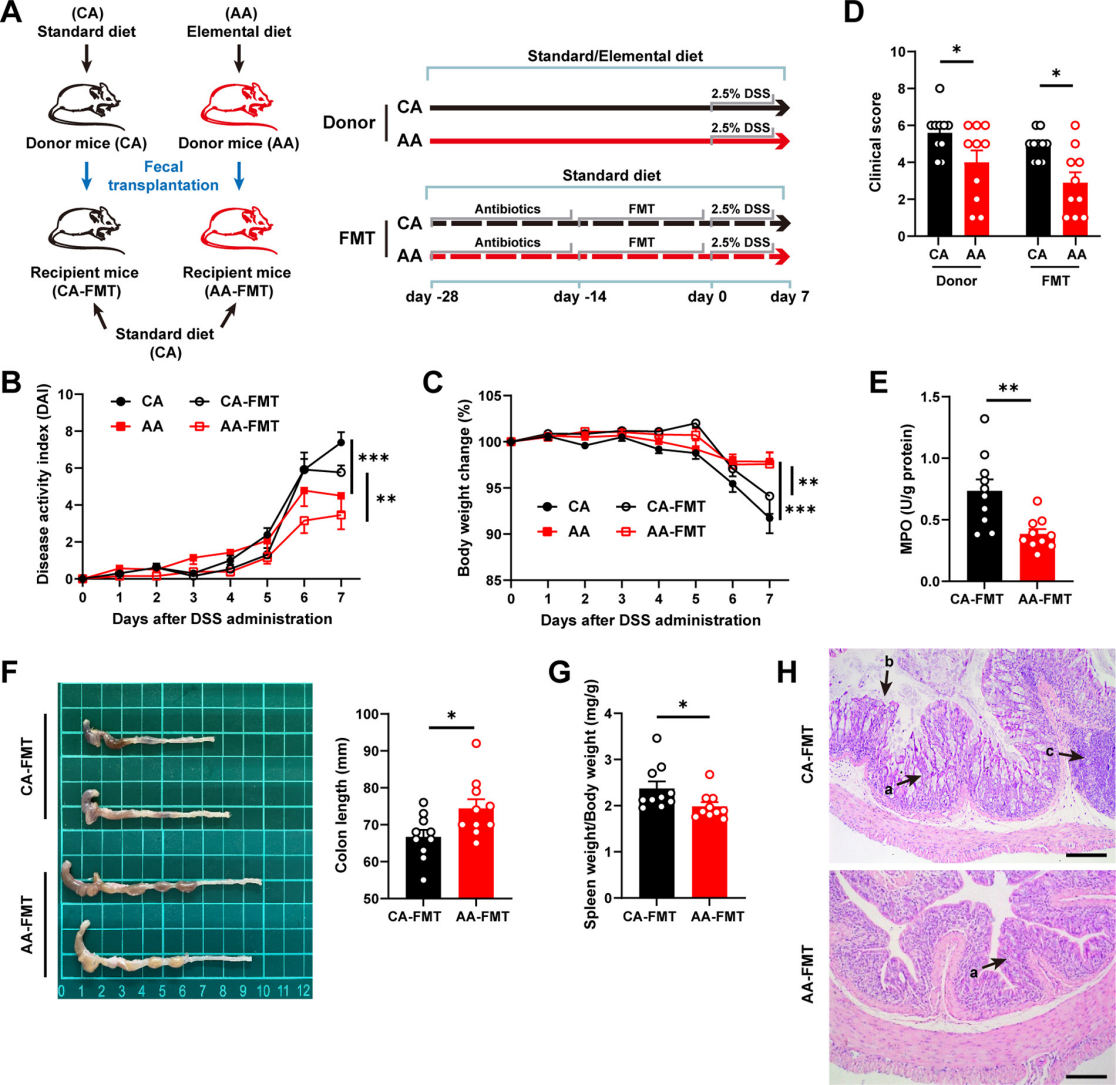
Prevention of colitis by the elemental diet is microbiota dependent. (A) Schematic illustration of the experimental design; (B) body weight; (C) disease activity index; (D) clinical score; (E) MPO activity in colon tissue; (F) colon length; (G) spleen weight (G); (H) H&E staining sections of colon tissue. Arrows show crypt deformation (a), epithelial damage (b), and infiltration of inflammatory cells in the submucosa (c). Data are means and SEM, and significance was examined by two-way ANOVA followed by Bonferroni’s multiple-comparison tests (B, C, and D) (*n* = 10) and Student’s *t* test (E, F, and G) (*n* = 10). *, *P* < 0.05; **, *P* < 0.01; ***, *P* < 0.001.

Next, we examined the effects of the ED on acute colitis induced by exposure to 2.5% DSS for 7 days. Compared with chronic colitis, the composition of gut microbiota is more stable in the acute-colitis model due to the reduced induction time. To confirm that the gut microbiota plays an important role in the anti-inflammatory effects of the ED, we performed fecal microbiota transplantation (FMT) experiments. Donor mice were fed AA or CA diets for 2 weeks. Recipient mice were treated with antibiotic cocktails for 10 days before gavage to deplete gut microorganisms (Fig. S3). After 14 days of fecal bacteria transplantation, mice were treated with DSS for 7 days to induce colitis (Fig. 5A).

10.1128/msystems.00883-22.7 FIG S3 Effect of an antibiotic cocktail on the gut microbiota in mice. (A) Concentration of DNA in feces before and after antibiotic treatment. (B) Relative content of fecal bacterial 16S rRNA. Data are means and SEM; significance was examined using two-way ANOVA followed by Bonferroni’s multiple-comparison test (*n* = 10). *, *P* < 0.05; **, *P* < 0.01; ***, *P* < 0.001. Download FIG S3, TIF file, 0.1 MB. Copyright © 2022 Zhang et al. 2022 Zhang et al. https://creativecommons.org/licenses/by/4.0/ This content is distributed under the terms of the Creative Commons Attribution 4.0 International license.

According to the results of 16S sequencing, there were differences in gut microbiota compositions between recipient mice and donor mice during FMT (Fig. S4), which was consistent with previous studies (30). Importantly, the gut microbes of mice in the CA receptor group (CA-FMT) and the AA receptor group (AA-FMT) group showed the same trend of change as the corresponding donor groups, indicating that FMT did shape the composition of gut microbiotas.

10.1128/msystems.00883-22.8 FIG S4 Changes in the overall gut microbiota of donor and recipient mice during FMT. (A) Principal-coordinate analysis (PCoA) of gut microbiota in donor and recipient mice before DSS induction. (B) PCoA of gut microbiotas in donor and recipient mice after DSS induction. (C and D) Differences for PC1 and PC2 among groups before (C) and after (D) DSS induction. Significance was examined using the Kruskal-Wallis test followed by Dunn’s multiple-comparison tests (*n* = 10). *, *P* < 0.05; **, *P* < 0.01; ***, *P* < 0.001. Download FIG S4, TIF file, 0.8 MB. Copyright © 2022 Zhang et al. 2022 Zhang et al. https://creativecommons.org/licenses/by/4.0/ This content is distributed under the terms of the Creative Commons Attribution 4.0 International license.

Compared with the donor CA mice, the donor AA mice had decreased DAI levels and increased body weight (Fig. 5B and C), suggesting that the ED ameliorated acute colitis in mice caused by a 7-day DSS induction. Compared with the CA-FMT mice, the AA-FMT mice had lower DAIs and longer colons, along with increased body weight, improved histopathology, and decreased MPO activity (Fig. 5B to H). The AA-FMT group had a lower clinical score (Fig. 5D), which was calculated based on the severity of diarrhea and gross rectal bleeding, but not body weight.

**FIG 6:**
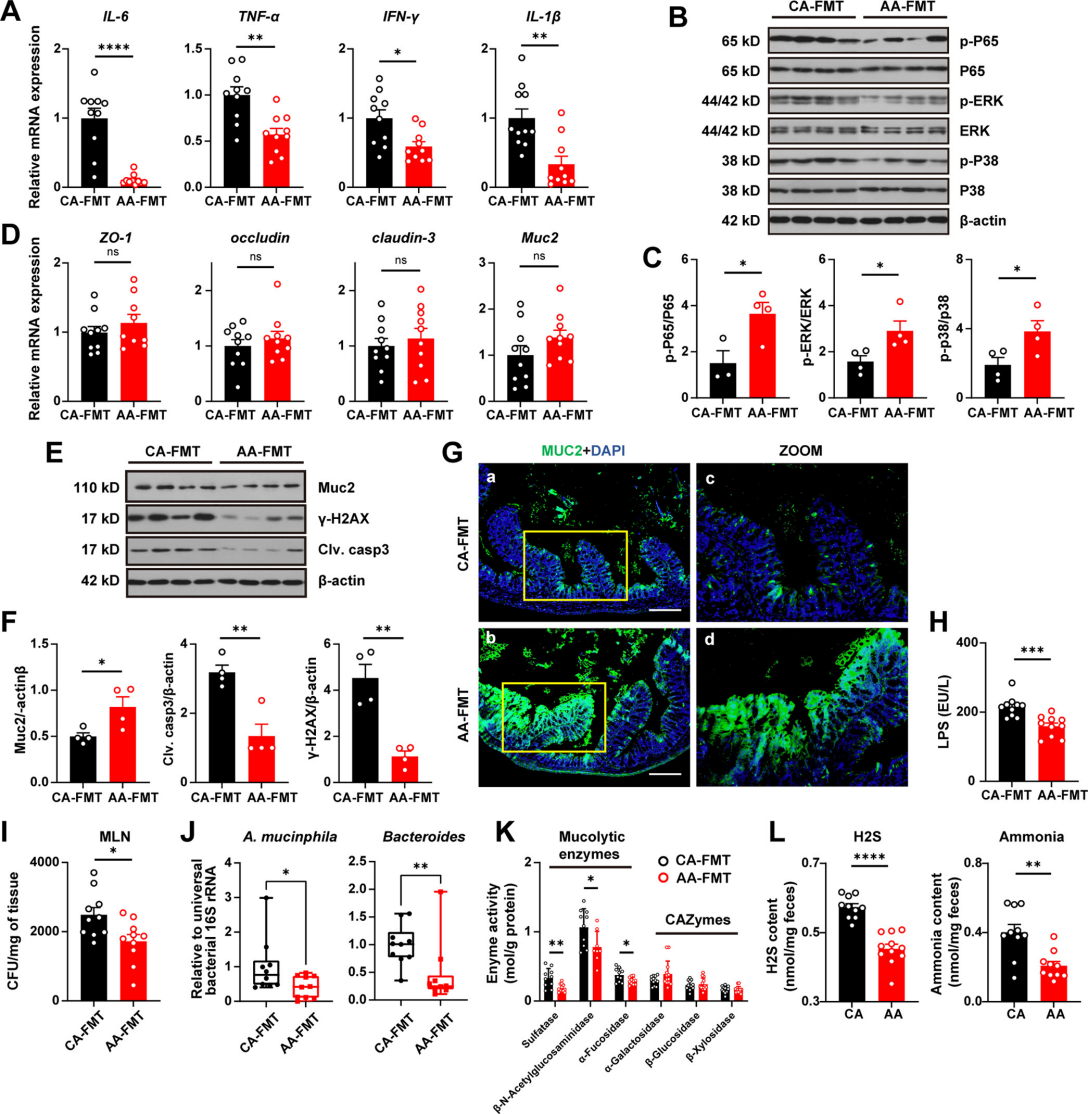
Fecal transplantation of an elemental diet-shaped microbiota alleviates erosion of the colonic mucus layer. (A) Relative mRNA expression of cytokines in colon tissue (A). (B and C) Expression levels of NF-κB and MAPK pathway-related proteins. (D) Relative mRNA expression of gut barrier-related proteins. (E and F) Expression levels of Muc2, gamma H2A histone family member X, and cleaved caspase-3 in colon tissue (E and F). (G) Immunofluorescence analysis of colon sections. Magnification, ×100. Bar = 200 μm. (H) LPS level in serum. (I) Quantification of bacterial translocation in the mesenteric lymph nodes. (J) Ratio of relative abundances of *A. muciniphila* and *Bacteroides* measured by qPCR. (K) Activities of mucolytic enzymes and carbohydrate-active enzymes. (L) Fecal content of H_2_S and ammonia. Data are means and SEM. Significance was examined by Student’s *t* test (A, C, D, F, H, I, K, and M) (*n* = 10) and the Mann-Whitney test (J) (*n* = 10). *, *P* < 0.05; **, *P* < 0.01; ***, *P* < 0.001; ****, *P* < 0.0001; ns, not significant.

In addition, the mRNA expression of inflammatory cytokines IL-6, TNF-α, IFN-γ, and IL-1β was decreased in the AA group (Fig. 6A). Western blot results showed that the phosphorylation level of NF-κB p65 in the AA-FMT group was significantly downregulated compared with the CA-FMT group, and the phosphorylation levels of Erk and p38 in the MAPK pathway were significantly reduced (Fig. 6B and C).

### Fecal transplantation of elemental diet-shaped microbiotas alleviated erosion of the colonic mucus layer.

Furthermore, we investigated the effect of fecal bacterial transplantation on the expression of intestinal barrier-related proteins in mice. The results showed that there were comparable differences in the protein and mRNA expression levels of occludin, ZO-1, and claudin-3 between the CA-FMT and AA-FMT groups (Fig. 6D). However, significant upregulation of Muc2 was observed in the AA-FMT group at the protein level (Fig. 6E and F). This result was further confirmed by immunofluorescence (Fig. 6G).

The level of serum LPS was also decreased in the AA-FMT group (Fig. 6H). Correspondingly, bacterial translocation into the mesenteric lymph nodes (MLN) was significantly inhibited in the AA-FMT group (Fig. 6I). Compared with the CA-FMT group, the expression levels of cleaved caspase-3, the executioner of apoptosis, and the DNA damage marker γ-H2AX in colon tissues of the AA-FMT group were significantly reduced (Fig. 6E and F).

We then measured the abundance of major mucin-degrading bacteria in the feces of recipient mice. According to the results of reverse transcription-PCR (RT-PCR), the relative abundance of *A. muciniphila* and *Bacteroides* was significantly decreased in the FMT-AA group (Fig. 6J). Correspondingly, the activities of mucolytic enzymes were decreased in the FMT-AA group (Fig. 6K), but the activities of CAZymes were not changed.

The elemental formula is thought to reduce food residues reaching the colon (22). To explore the underlying mechanism by which the ED affects the microbial community, we measured the content of protein degradation products in feces (Fig. 6L). Notably, donor mice fed the AA diet had significantly lower levels of H_2_S and ammonia in the feces than the mice fed the CA diet.

These observations indicate that the ED prevents colitis includes by reducing the number of mucus-degrading bacteria, thereby inhibiting mucus degradation and suppressing apoptosis and inflammation caused by bacterial translocation. The reduction of proteins entering the large intestine for fermentation plays a key role in the alteration of the gut microbiota.

### Prevention of colitis by the elemental diet is ineffective in pseudogermfree mice.

**FIG 7:**
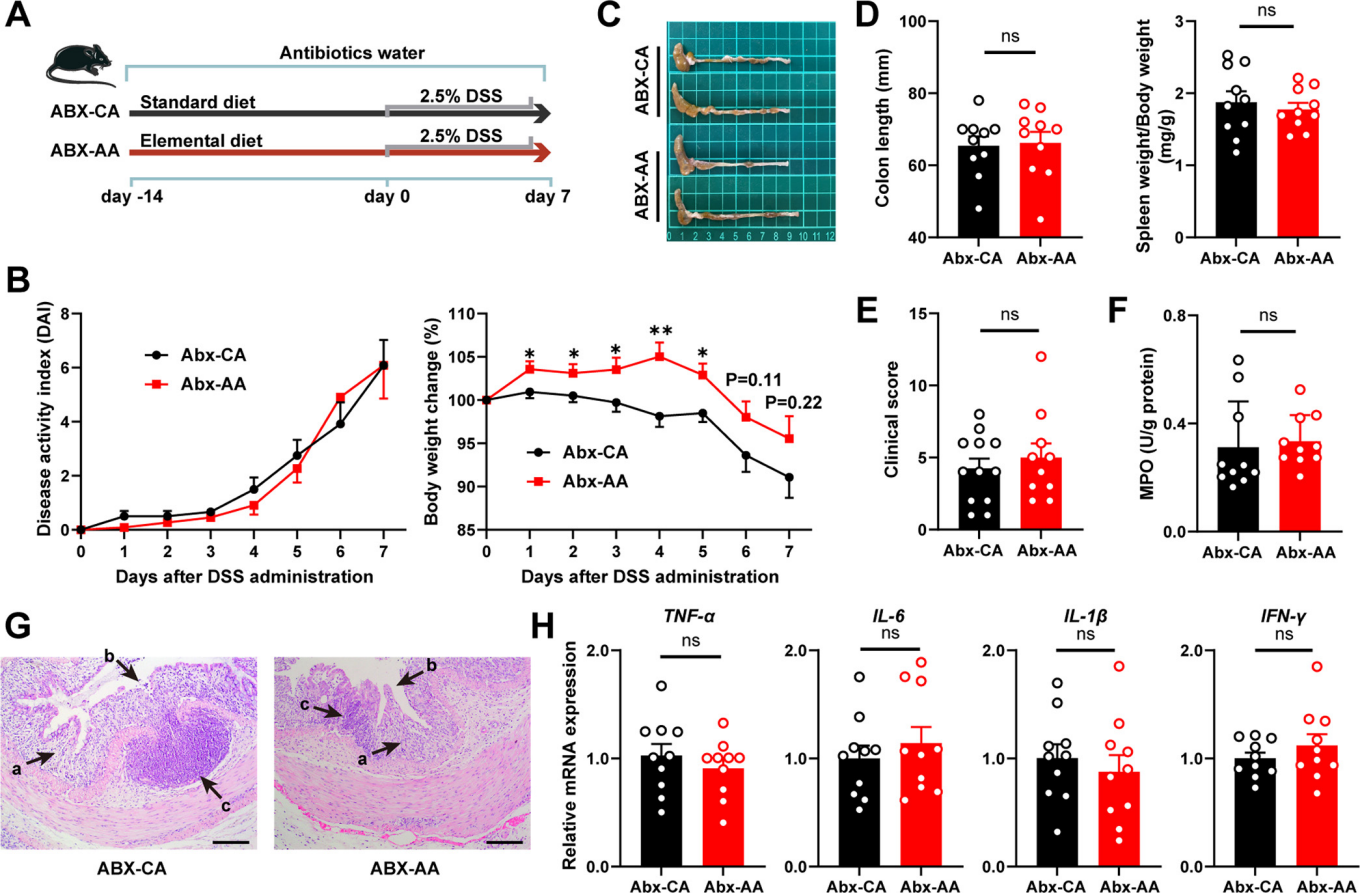
Prevention of colitis by the elemental diet is ineffective in antibiotic-treated pseudogermfree mice. (A) Schematic illustration of the experimental design. (B) Body weight and disease activity index. (C) Colon photos. (D) Colon length and spleen weight. (E) Clinical score. (F) MPO activity in colon tissue. (G) H&E-stained sections of colon tissue. Arrows show crypt deformation (a), epithelial damage (b), and infiltration of inflammatory cells in the submucosa (c). (H) Relative mRNA expression of cytokines (H). Data are means and SEM, and significance was examined by Student’s *t* test (*n* = 10). *, *P* < 0.05; **, *P* < 0.01; ns, not significant.

Last, we compared the effects of the ED (ABX-AA, antibiotics-treated AA mice) and the casein diet (ABX-CA) on colitis in pseudogermfree mice (Fig. 7A). Mice were given a broad-spectrum antibiotic cocktail. Bacterial load and fecal DNA concentrations were determined to ensure that the gut microbiota was depleted before DSS induction.

During the DSS induction period, the body weight was increased in the ABX-AA mice compared with the ABX-CA mice (Fig. 7B). However, the values of DAI and clinical score were comparable between the two groups (Fig. 7B and E). In terms of morphology and histopathology, the colon length, edema, crypt morphology, epithelial damage, and inflammatory cell infiltration in the submucosa were comparable in the two groups (Fig. 7C, D, and G). There was no difference in the activity of inflammation marker MPO (Fig. 7F) in the colon tissue. Similarly, no difference was observed in the expression of the proinflammatory cytokines IL-1β, IL-6, TNF-α, and IFN-γ and the mRNA expression of ZO-1, occludin, claudin, and MUC2 (Fig. 7H and Fig. S5). The above results indicate that the ED has no anti-inflammatory effect in mice with deficient gut microbiotas.

10.1128/msystems.00883-22.9 FIG S5 Relative mRNA expression of gut barrier-related proteins in antibiotic-treated pseudogermfree mice. Data are means and SEM; significance was examined with Student’s *t* test (*n* = 10). *, *P* < 0.05; **, *P* < 0.01; ***, *P* < 0.001. Download FIG S5, TIF file, 0.2 MB. Copyright © 2022 Zhang et al. 2022 Zhang et al. https://creativecommons.org/licenses/by/4.0/ This content is distributed under the terms of the Creative Commons Attribution 4.0 International license.

## DISCUSSION

IBD is a chronic and relapsing inflammatory disease with complex pathogenesis. It is well known that disruption of the gut microbiota is an important factor in the progression of IBD (31). A diet that forms a healthy gut microbial community is an efficient way to modulate host immune response (32), which provides a new strategy for the dietary treatment of IBD. ED therapy has been used as a primary treatment for IBD patients in many parts of the world (18). However, the efficacy of elemental and polymeric formulations remains controversial. The mechanism of ED therapy might be related to alterations in the physical form of the diet, digestion and absorption, and the gut microbiota, but the underlying mechanism is not clearly identified. In this study, we compared the effect of an amino acid-enriched ED and an intact-protein-enriched polymeric diet on the progression of colitis in mice. We observed increased body weight, decreased DAI, increased colon length, and reduced colon lesions in the ED-treated mice. In conclusion, the ED was more effective not only in preventing the progression of chronic colitis but also in preventing the development of acute intestinal inflammation.

Mucus is the first line of defense for intestinal self-protection, physically isolating harmful bacteria (10). Intestinal mucus degradation can lead to colitis in mice (33). Muc2 is the main component of the colonic mucus layer, which forms the skeleton of the mucus (9). It was found that Muc2 knockout in mice could cause inflammation that eventually leads to colorectal cancer (34). Therefore, Muc2 is considered a potential therapeutic target for IBD (35). Notably, more than 80% of the mass in mucin is O-glycans, mainly linked by O-glycosidic bonds, and the C and N termini are linked by disulfide bonds (9). Some intestinal bacteria utilize mucin as their main carbon source. They can secrete glycosidase and sulfatase to degrade mucin (12). In this study, we found that the degradation of colonic mucus was effectively inhibited in mice fed the ED. Notably, there was no significant difference in the mRNA expression of *Muc2* between the ED group and the casein group, and there was no significant difference in the expression of barrier proteins occludin, claudin, and ZO-1. However, at the protein level, the content of Muc2 in the ED group was significantly increased. The results indicated that the change of Muc2 was not due to the regulation of mRNA expression. It was observed that the activities of mucolytic enzymes were significantly decreased by ED treatment, and this change depends on the gut microbiota. Taken together, these observations suggest that the increased content of Muc2 could be the underlying means by which the ED improved IBD and that this Muc2 content is mediated by intestinal mucolytic bacteria.

The gut microbiota is an important mediator between environmental factors and host health. Short-term changes in diet have been shown to rapidly alter the human gut microbiota (36). The imbalance of gut microbiomes is the main pathogenesis of IBD. The results of our microbiota analysis revealed why the ED inhibited the degradation of colonic mucin in mice. *Akkermansia* has received increasing attention in recent years, but its health effects remain controversial. Some studies have found that *Akkermansia* fecal transplantation can extend life span in mice with progeria (37) and alleviate metabolic diseases such as obesity and diabetes in mice (38). But it is worth noting that numerous studies have shown its negative effects when the host is in a pathological state. For example, the increase of *Akkermansia* in the colon following vitamin deficiency enhances mucolysis, which leads to intestinal barrier dysfunction and enhances pathogen susceptibility (6). Similarly, *Akkermansia* is found to be abundant in mice with high levels of intestinal inflammation (32), and acts as a pathogen to promote colitis in IL-10^−/−^ mice (39). More importantly, clinical research has revealed that IBD patients have higher levels of mucolytic bacteria (11), of which *Akkermansia* and *Bacteroides* are the main members (6). In this study, the relative abundance of *Akkermansia* and *Bacteroides* decreased after ED intervention. This phenomenon was also observed after fecal bacterial transplantation. Correspondingly, microbial translocation toward intestinal lymph nodes was inhibited in both recipient and donor mice. These results strongly supported our view that the ED can reduce the abundance of mucolytic bacteria and prevent harmful microorganisms from invading intestinal epithelial cells. Future studies should compare the differences in the expression of mucolytic enzymes in different strains. Specific mucolytic strains should be isolated to identify mucolytic-enzyme-related genes and to explore their role in intestinal inflammation.

In addition to mucolytic bacteria, we also found changes in the abundance of other bacteria. ED treatment increased the relative abundance of *Muribaculaceae* and *Faecalibaculum* in mouse feces and decreased the relative abundance of *Alistipes*. In terms of pathogenicity, there is evidence that *Alistipes*, a newer subclass of *Bacteroidetes*, is positively correlated with diarrhea and abdominal pain and is pathogenic in colorectal cancer (37). Therefore, the decrease in the relative abundance of intestinal *Alistipes* in mice fed the ED is related to the reduction of susceptibility to colitis. *Muribaculaceae* have multiple functions in degrading complex carbohydrates (40). Similar to *Bifidobacterium* and *Lactobacillus*, *Faecalibaculum* can produce lactic acid and short-chain fatty acids and has anti-colon cancer effects (41). It protects the stability of the intestinal environment and prevents pathogens from colonizing in the intestinal epithelium, which has health benefits. Therefore, the higher abundance of *Muribaculaceae* and *Faecalibaculum* and lower abundance of *Alistipes* in the intestines of mice fed the ED may also help reduce the pathogenesis of colitis. Furthermore, we observed decreased content of H_2_S and ammonia in the feces of mice treated with the ED. The result suggests that the modulation of gut microbiota is likely to result from a reduction in the fermentation of unabsorbed proteins.

Endogenous endotoxins are produced by gut microbes and enter the blood circulation with the disruption of the gut barrier (42). The LPS-LBP complex binds to Toll-like receptor 4 (TLR4) and activates MAPK and NF-κB signaling pathways (43). Previous studies have shown that colitis can be alleviated by inhibiting the NF-κB or MAPK signaling pathway and reducing the release of downstream proinflammatory cytokines (44, 45). In this study, we found that the ED significantly reduced the serum LPS level in mice with colitis. The phosphorylation levels of NF-κB P65, P38, and ERK were significantly downregulated, indicating that the ED exerts an anti-inflammatory effect by preventing LPS from passing the intestinal epithelial barrier and inhibiting the NF-κB/MAPK inflammatory signal pathway. Moreover, NLRP3 inflammasome has been found to activate the expression of IL-1β and IL-18, thereby promoting the progression of IBD (46). IL-12 activates Th1 differentiation and IFN-γ release to promote intestinal mucosal inflammation (47). IL-6 and IL-23 can stimulate Th17 cells to produce IL-17 family cytokines (48), which are important regulators of intestinal mucosal inflammation. In this study, the ED decreased the expression of IL-1β, IL-12, and IFN-γ. The expression of IL-12 and IL-23 was also downregulated, but there was no difference in the expression of IL-17. The results indicate that the ED may exert its protective effect through the inhibition of NLRP3 inflammasome activation and Th1 differentiation, rather than Th17 differentiation.

There are several limitations to this study. First, antibiotic treatment cannot completely remove the intestinal microbiota. Thus, the possibility that some of the residual microbiota affect intestinal inflammation cannot be ruled out. Second, FMT did not adequately replicate the intestinal flora of donor mice. Therefore, the role of some specific microorganisms may have been overlooked in the receptor mice.

### Conclusion.

In conclusion, the ED has better preventive effects on IBD than polymeric diets. By utilizing fecal microbiota transplantation and pseudosterile animals, we convincingly demonstrated that the gut microbiota plays a critical role in the effects of the ED. An ED can reduce the abundance of mucus-degrading bacteria, thereby inhibiting the mucus layer disruption and preventing harmful microbes from invading intestinal epithelium. This study may provide new insights into the gut microbiota-diet interaction in human health.

## MATERIALS AND METHODS

### Materials.

DSS (molecular weight [MW], 36 to 50 kDa) was obtained from MP Biomedicals LLC (Santa Ana); TRIzol reagent (Thermo Fisher Scientific), a LunaScript SuperMix kit (New England BioLabs), and SYBR qPCR master mix (ChamQ Universal) were used for RT-qPCR analysis. Antibodies (β-actin, p38, p-p38, JNK, p-JNK, Erk, p-Erk, p-p65, p65, occludin, ZO-1, claudin-1, MUC-2, caspase-3, and γ-H2AX) were purchased from Cell Signaling Technology; the MPO kit and ELISA kits for LPS were purchased from Nanjing Jiancheng Bioengineering Institute. A fecal DNA isolation kit was purchased from Qiagen.

### Animals and treatments.

Specific-pathogen-free (SPF) ICR mice (6 weeks old) were purchased from Beijing Charles River Laboratory Animal Technology Co., Ltd. To reduce experimental error in colitis-related indicators, only male mice were used. The mice were placed in an environment with controlled temperature (25 ± 1°C) and relative humidity (50% ± 5%) with free access to food and water. The adaptation period was 1 week. All animal experimental protocols were approved by the Institutional Animal Care and Use Committee of Nankai University and carried out following the national ethical guidelines for laboratory animals (permission number SYKX 2019-0001).

**(i) Effects of the ED on DSS-induced colitis in normal mice.** One week after acclimation, the mice were weighed and randomly divided into four groups: the casein (CA) diet group, the amino acid-based (AA) ED group, the CA-DSS group, and the AA-DSS group, with 12 mice in each. The CA group mice were fed an AIN-93G standard diet. In the AA diet, the casein in AIN-93G was replaced with amino acids of the same composition. All diets were adjusted to the same energy level. The diet composition is listed in Table 1, and the amino acid profile of casein is shown in Table S1.

**TABLE 1:** Composition of experimental diets

Ingredient	Concn (g/kg) in diet	
	CA	AA
Corn starch	397.5	399.1
Maltodextrin	132	132
Cellulose	50	50
Soybean oil	70	70
Mineral mix	3.5	3.5
Sodium bicarbonate	0	7.5
Vitamin mix	10	10
Choline bitartrate	2.5	2.5
l-Cystine	3	0
Amino acid mix	0	192
Casein	200	0

10.1128/msystems.00883-22.2 TABLE S1 Amino acid profiles of casein. Download Table S1, DOCX file, 0.02 MB. Copyright © 2022 Zhang et al. 2022 Zhang et al. https://creativecommons.org/licenses/by/4.0/ This content is distributed under the terms of the Creative Commons Attribution 4.0 International license.

After all mice had been fed a specific diet for 2 weeks, two groups were treated with DSS (1.5%) in drinking water for 3 cycles (5 days/cycle, with a 7-day recovery after each of the first 2 cycles) (Fig. 1A). Food consumption, body weight, and DAI were recorded regularly. At the end of the third round of DSS, mouse feces were collected, and the mice were euthanized to obtain serum. Colon tissues were immediately collected, weighed, photographed, and stored at −80°C.

**(ii) Effects of fecal microbiota transplantation from ED-fed mice on colitis.** To evaluate the effect of the ED on acute colitis and to maintain the stability of the gut microbiota during colitis induction, an acute-colitis model induced by a 7-day DSS exposure was used. The donor mice were fed a casein diet (CA) or the ED (AA) for 2 weeks (Fig. 5A). Fresh feces from CA and AA groups were collected and suspended in phosphate-buffered saline (PBS) (10% [wt/vol]). Fecal homogenates were centrifuged at 800 × *g* for 5 min, and the supernatant was collected. Fecal supernatants were administered by oral gavage to antibiotic-treated pseudogermfree mice. To deplete the microbiota, the recipient mice were treated with an antibiotic cocktail (metronidazole, 1 g/L; neomycin, 1 g/L; vancomycin, 500 mg/mL; ampicillin, 1 g/L) for 2 weeks. Depletion of the microbiota was confirmed by bacterial colony assays and real-time PCR analysis of universal bacterial 16S rRNA as described above. Two weeks after fecal transplantation, mice were administered 2.5% DSS to induce colitis.

**(iii) Effects of the ED on colitis in antibiotic-treated pseudogermfree mice.** To deplete the intestinal flora, mice were treated with water containing a mixture of antibiotics (metronidazole, 1 g/L; neomycin, 1 g/L; vancomycin, 500 mg/mL; ampicillin, 1 g/L) for 14 days (Fig. 7A). To observe bacterial depletion, fecal DNA was isolated, and the universal bacterial 16S rRNA gene was analyzed by real-time qPCR. Additionally, mice were treated with antibiotics and 2.5% DSS for 7 days and then sacrificed. Mice were divided into two groups, one fed with the casein diet (ABX-CA) and one with the ED (ABX-AA).

### Disease activity index and clinical score.

Disease activity index (DAI) scores were conducted periodically to assess the severity of colitis. Scoring indicators included body weight loss, stool consistency, and gross bleeding. Each indicator was scored as follows: body weight loss was scored as 0 (none), 1 (1 to 5%), 2 (5 to 10%), 3 (10 to 20%), and 4 (>20%); stool consistency was scored as 0 (normal), 1 and 2 (loose stool), and 3 and 4 (diarrhea); and stool blood was scored as 0 (negative), 1 and 2 (blood), and 3 and 4 (gross bleeding) (49). To avoid the influence of body weight change on the statistical analysis of DAI, the clinical score was calculated based on the severity of diarrhea and gross rectal bleeding (50).

### Histopathological analysis.

The distal colon tissue (5 mm) was fixed in 10% formalin solution for 24 h, embedded in paraffin, sectioned, and stained with hematoxylin and eosin (H&E). Blind photographs were taken of each colon sample under a microscope at ×100 magnification. Histological scores were assigned according to the severity of crypt depletion and distortion (0 to 3), the degree of inflammatory infiltration (0 to 4), and the area of involvement (0 to 4) (51).

### Real-time qRT-PCR.

Total RNA was extracted from the colon tissues (50 to 100 mg) using TRIzol reagent (Thermo Fisher Scientific). The total RNA was quantified and then reverted to cDNA. Real-time PCR was measured using the SYBR green master mix (New England Biolabs [NEB]). Cycle threshold (*C_T_*) was used to calculate the relative expression of the target gene by the 2^−ΔΔ^*^CT^* method. Primers for mRNA expression measurement are listed in Table S2.

10.1128/msystems.00883-22.3 TABLE S2 Primers for mRNA expression measurement in RT-qPCR. Download Table S2, DOCX file, 0.02 MB. Copyright © 2022 Zhang et al. 2022 Zhang et al. https://creativecommons.org/licenses/by/4.0/ This content is distributed under the terms of the Creative Commons Attribution 4.0 International license.

### Western blotting and ELISA.

Colon tissue was triturated with an inhibitor cocktail (Beyotime, Beijing, China; 1:50 [vol/vol]) in radioimmunoprecipitation assay (RIPA) lysis buffer to obtain a protein solution. After centrifugation, the protein concentration was determined with a bicinchoninic acid (BCA) assay kit. The samples were separated by 12% SDS-PAGE, transferred to the nitrocellulose filter (NC) membrane, and incubated with antibodies. Membranes were washed three times with Tris-buffered saline–Tween (TBST) and developed using the Pierce enhanced chemiluminescence (ECL) Western blotting kit (32106; Thermo Fisher). Antibodies were diluted 1:1,000. Horseradish peroxidase (HRP)-conjugated secondary antibodies (1:4,000 dilution; 31460; Thermo Fisher) were incubated for 1 h at room temperature for protein detection.

The level of lipopolysaccharide (LPS) in serum was detected with an enzyme-linked immunosorbent assay (ELISA) kit (Nanjing Jiancheng Bioengineering Institute). The activity of MPO in colon tissue was determined with a commercially available kit (Nanjing Jiancheng Bioengineering Institute).

### Immunofluorescence analysis.

Paraffin-embedded samples were sectioned. The prepared sections were fluorescently stained via section dewaxing, protease K treatment, denaturation, hybridization, sealing, DAPI (4′,6-diamidino-2-phenylindole) restaining, and anti-fluorescent quencher sealing. The experimental procedure was protected from light, and then a microscopic examination was carried out. Three fields of view were randomly selected for each colon sample under ×100 and ×200 magnification.

### 16S rRNA high-throughput sequencing and bioinformatics analysis.

High-throughput sequencing and bioinformatics analysis were performed according to our previous method (52) and detailed methods are presented in the supplemental material, Text S1.

10.1128/msystems.00883-22.1 TEXT S1 16S rRNA high-throughput sequencing and bioinformatics analysis. Download Text S1, DOCX file, 0.02 MB. Copyright © 2022 Zhang et al. 2022 Zhang et al. https://creativecommons.org/licenses/by/4.0/ This content is distributed under the terms of the Creative Commons Attribution 4.0 International license.

### Determination of specific bacteria by qPCR.

Microbial DNA was extracted from the fecal samples using the fecal DNA isolation kit (Qiagen). The abundance of the mucolytic bacteria *Akkermansia*, *Bacteroides*, and species of *Bacteroides* in feces was further determined by qPCR by the 2^−ΔΔ^*^CT^* method (53, 54). The relative abundance of *Akkermansia* and *Bacteroides* was normalized to total bacteria and presented as the ratio between groups. Primers used for bacterial 16S rRNA gene detection were evaluated as previously described (6, 55, 56) and are listed in Table S3.

10.1128/msystems.00883-22.4 TABLE S3 Primers for the measurement of bacterial 16S rRNA genes by qPCR. Download Table S3, DOCX file, 0.02 MB. Copyright © 2022 Zhang et al. 2022 Zhang et al. https://creativecommons.org/licenses/by/4.0/ This content is distributed under the terms of the Creative Commons Attribution 4.0 International license.

### Determination of mucus-degrading enzyme activity.

The activity of bacteria-derived mucus-degrading enzymes and carbohydrate-active enzymes were determined according to the previously described method with minor modifications (55). Approximately 50 mg of feces was homogenized in enzyme analysis buffer (50 mM Tris, 100 mM KCl, 10 mM MgCl_2_; pH 7.26). One hundred microliters of 12% Triton X-100 buffer, lysozyme, and 10 mg of DNase and protease inhibitor (Roche) were added to the mixture and homogenized on ice to obtain a fecal suspension. The suspension was centrifuged at 10,000 × *g* for 10 min, and the obtained supernatant was used for the enzyme activity assay. Ten microliters of sample was mixed with 150 μL of a 10 nM substrate solution, including 4-nitrophenyl-2-acetamino-2-deoxy-β-d-glucopyranoside, 4-nitrophenylsulfate, 4-nitrophenyl-α-l-fucopyranoside, 4-nitrophenyl-α-d-galactopyranoside, 4-nitrophenyl-β-d-glucopyranoside, and 4-nitrophenyl-β-d-xylopyranoside prepared in the same assay buffer. The mixtures were incubated at 37°C for 2 h, and the absorbance at 405 nm was recorded. A standard curve was established with 4-nitrophenol.

### Assessment of bacterial translocation in mesenteric lymph nodes.

Microbial translocation in the mesenteric lymph nodes was detected according to the method previously described (57). Under anaerobic conditions, MLN were collected aseptically and homogenized in BBL Mycoflask thioglycolate (liquid) prepared medium (BD Diagnostic Systems). Gifu medium agar plates containing the contents of the BBL Mycoflask thioglycolate were incubated in an anaerobic incubator at 37°C for 48 h. CFU were counted, and the concentration per milligram of tissue was determined.

### Determination of fecal content of H_2_S and ammonia.

To determine the fecal content of H_2_S and ammonia, 50 mg of feces was homogenized in 500 μL of 0.1 M HCl. The samples were centrifuged at 12,000 × *g* for 20 min. The concentration of H_2_S was determined by the *N*,*N*-dimethyl-*p*-phenylenediamine sulfate method (58). The concentration of ammonia was determined by the phenol-hypochlorite method (59).

### Statistical analysis.

Statistical analysis was performed using GraphPad Prism 8.0. Data are presented as means and standard errors of the means (SEM). Differences among groups were compared by two-way analysis of variance (ANOVA) followed by Bonferroni’s *post hoc* test. Differences between two groups were analyzed by two-tailed unpaired Student’s *t* test or the Mann-Whitney test. *P* values of <0.05 were considered statistically significant.

### Data availability.

The data were deposited in the NCBI Sequence Read Archive with the accession numbers PRJNA841685 and PRJNA877839.
