# Supplementary figures and images for: An Elemental Diet Enriched in Amino Acids Alters the Gut Microbial Community and Prevents Colonic Mucus Degradation in Mice with Colitis
Source: mSystems. 2022 Dec 5;7(6):e00883-22. doi: 10.1128/msystems.00883-22 (PMC9765100; doi:10.1128/msystems.00883-22)

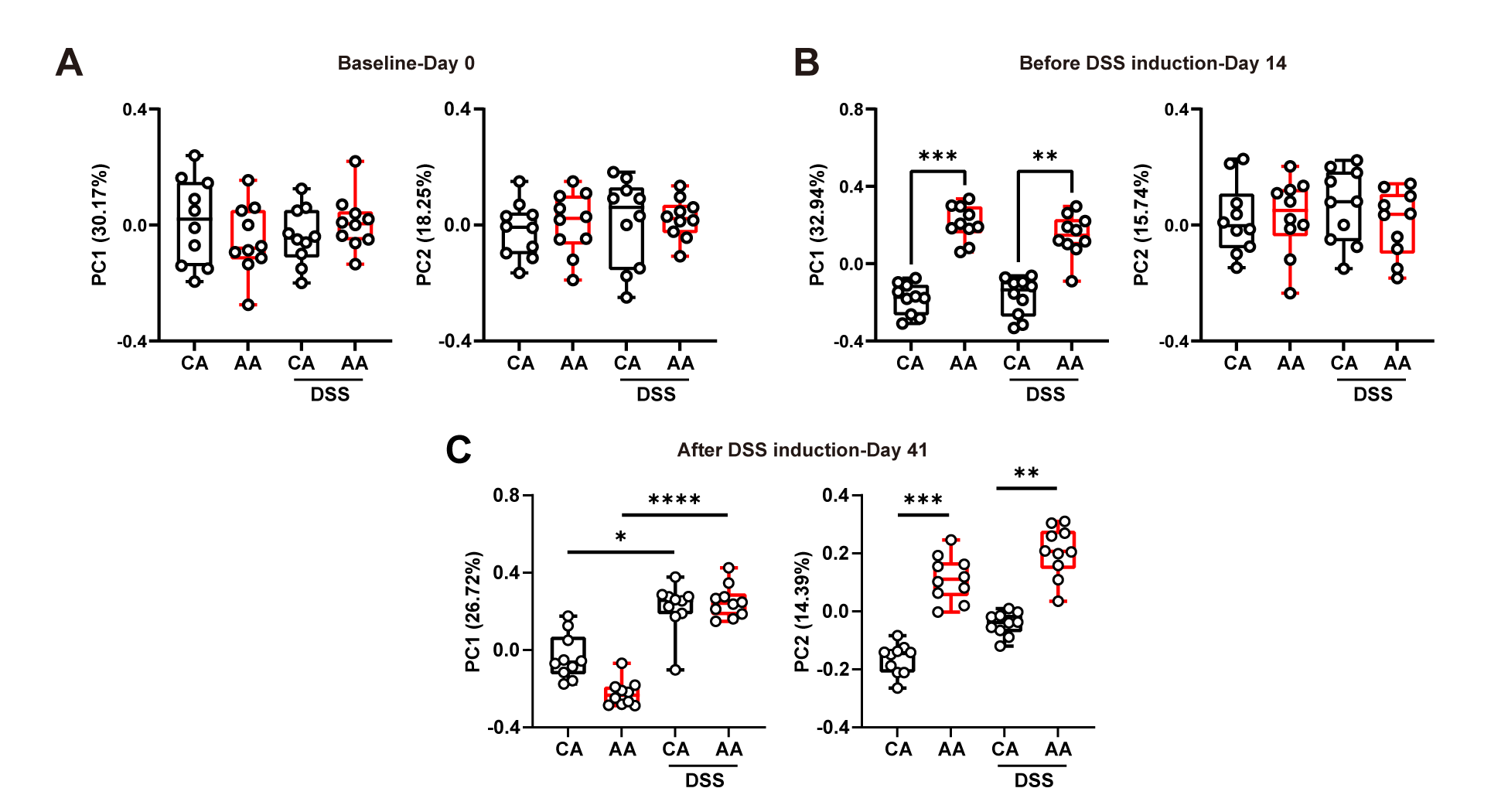

Supplement: FIG S1 [file msystems.00883-22-s0005.tif]

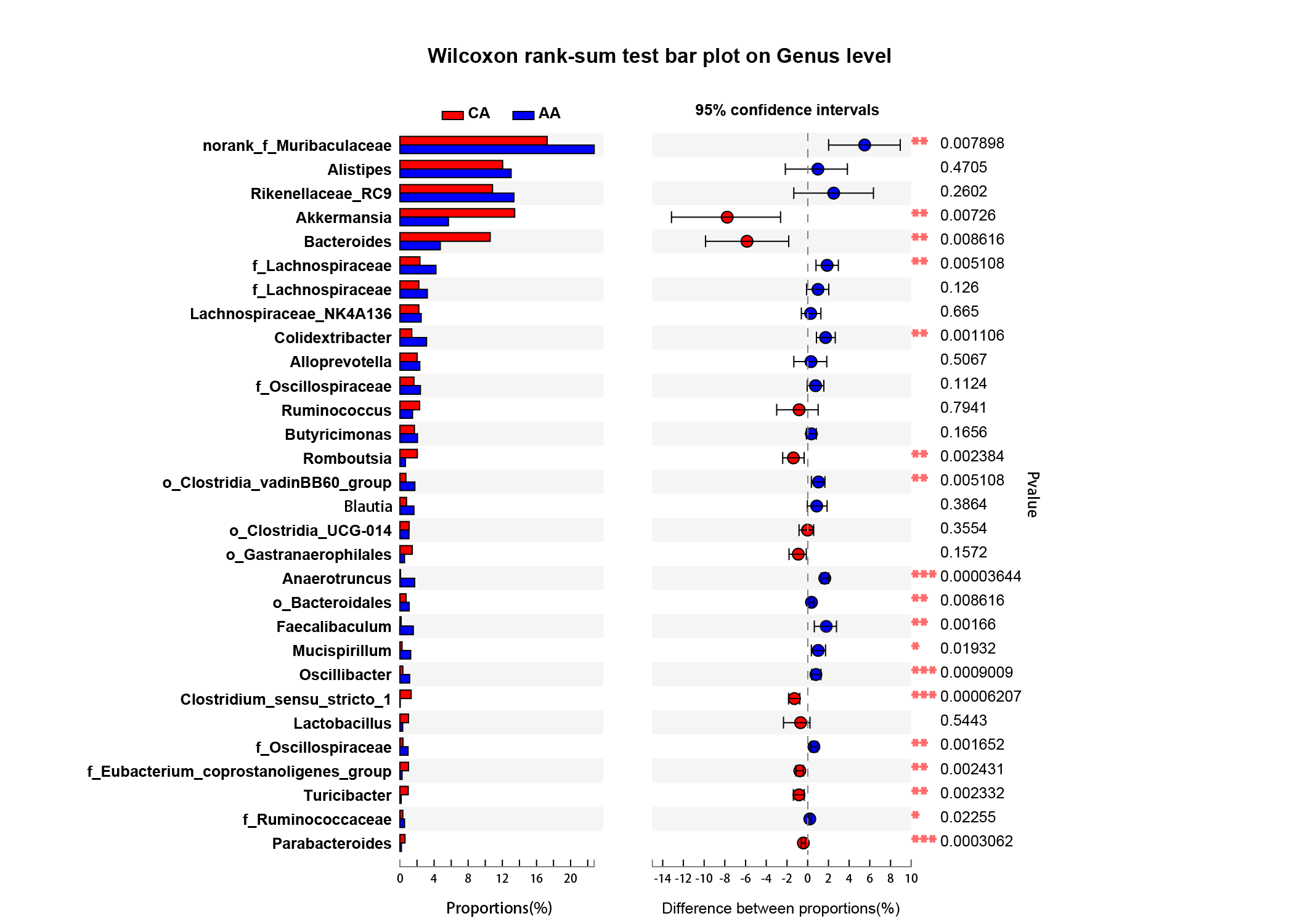

Supplement: FIG S2 [file msystems.00883-22-s0006.tif]

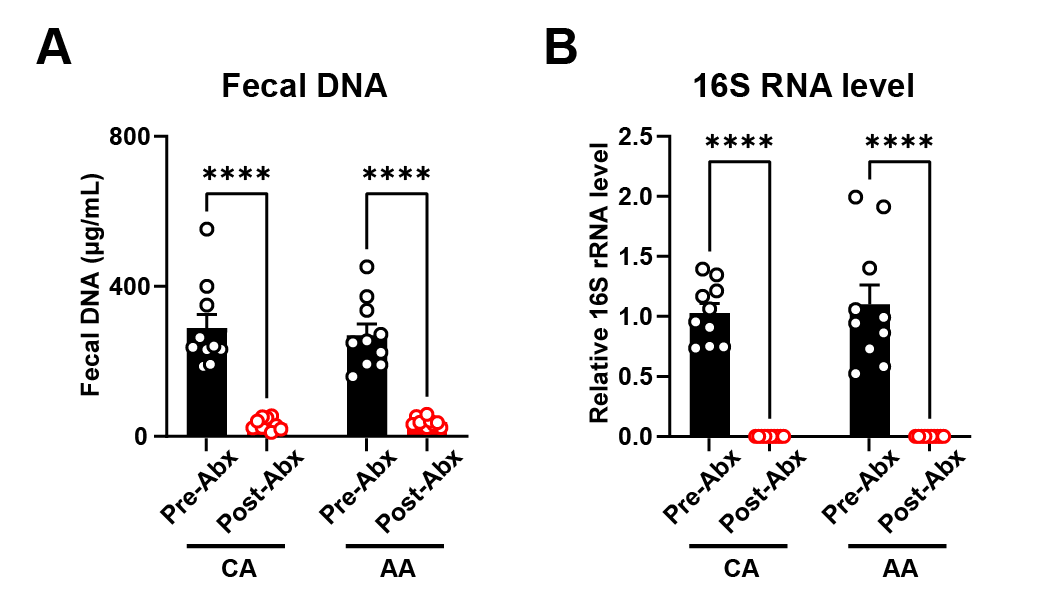

Supplement: FIG S3 [file msystems.00883-22-s0007.tif]

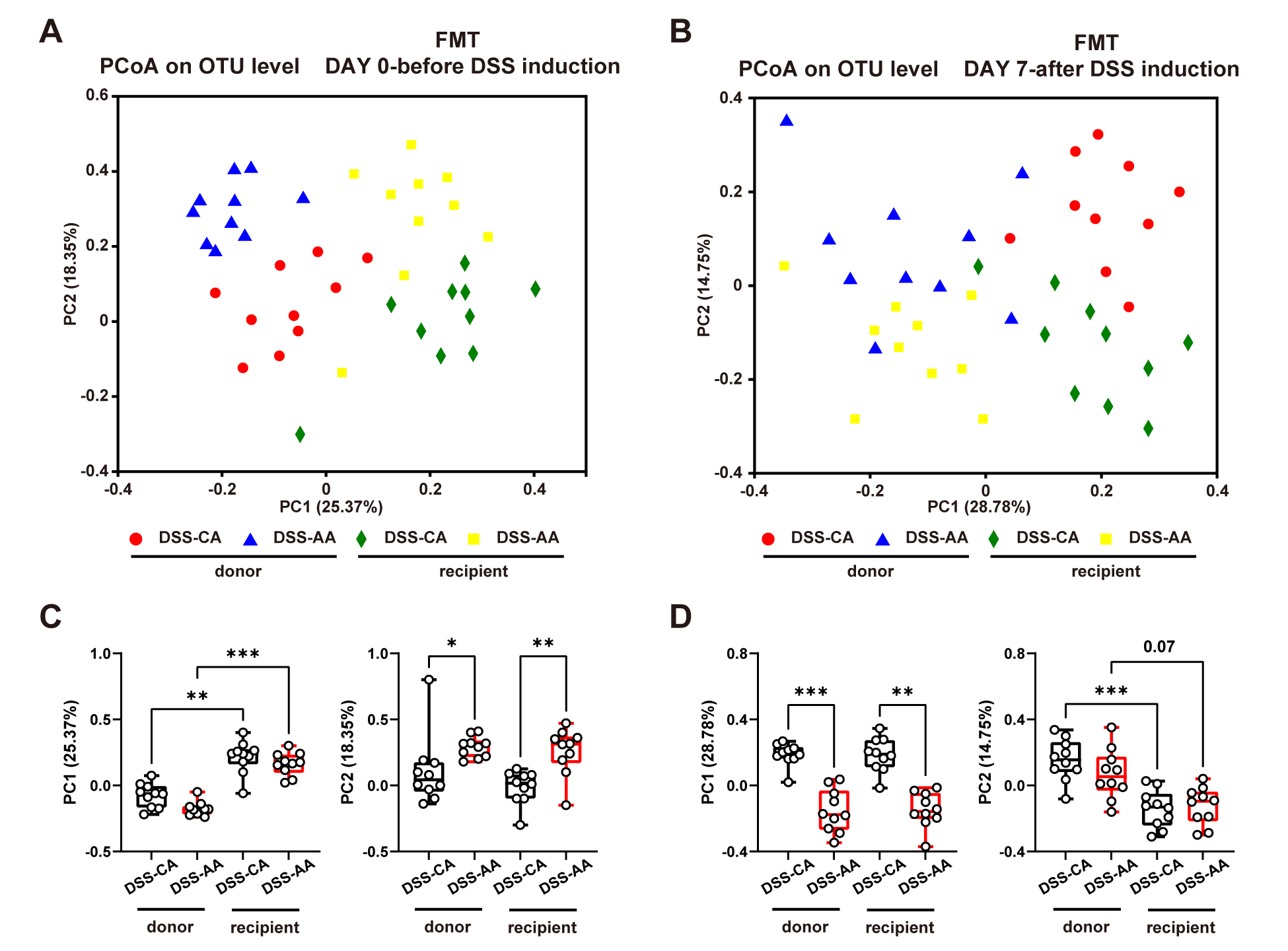

Supplement: FIG S4 [file msystems.00883-22-s0008.tif]

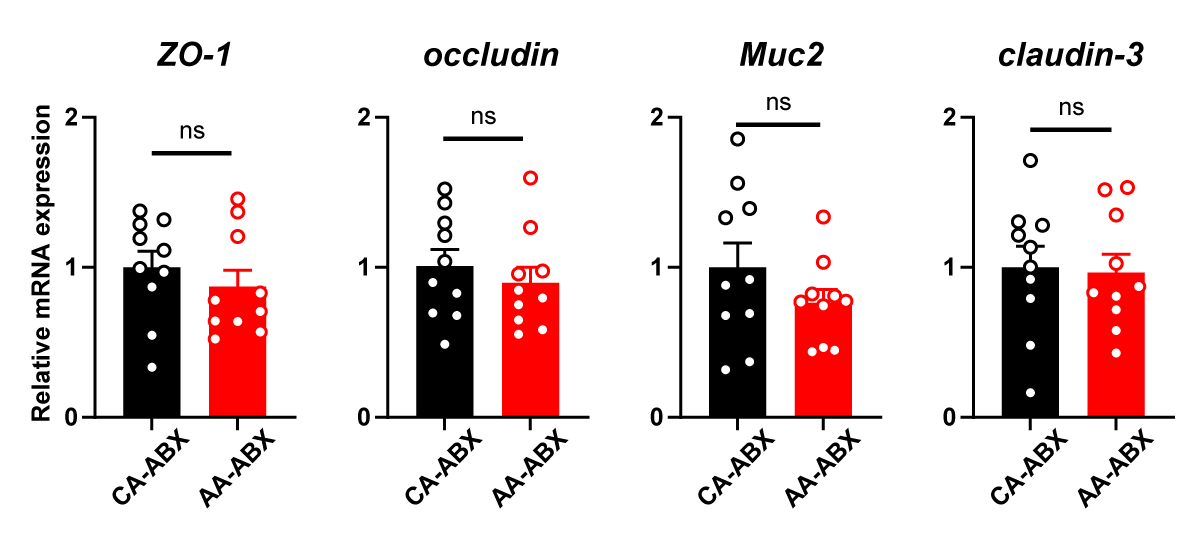

Supplement: FIG S5 [file msystems.00883-22-s0009.tif]
