# Supplementary material for: An Elemental Diet Enriched in Amino Acids Alters the Gut Microbial Community and Prevents Colonic Mucus Degradation in Mice with Colitis
Source: mSystems. 2022 Dec 5;7(6):e00883-22. doi: 10.1128/msystems.00883-22 (PMC9765100; doi:10.1128/msystems.00883-22)
Supplement: TABLE S1 [file msystems.00883-22-s0002.docx]

**Table S1.**

| Amino Acid Profile | |
| --- | --- |
| Essential Amino Acids | Content (%) |
| Isoleucine | 4.93 |
| Leucine | 8.75 |
| Lysine | 7.45 |
| Methionine | 2.79 |
| Phenylalanine | 4.84 |
| Threonine | 4.00 |
| Tryptophan | 1.21 |
| Valine | 6.24 |
| Total EAA | 40.22 |
|  |  |
| Non-Essential Amino Acids | Content (%) |
| Histidine (Essential for Infants) | 2.61 |
| Alanine | 2.79 |
| Arginine | 3.54 |
| Aspartic acid | 6.61 |
| Cysteine/cystine | 0.65 |
| Glutamic acid | 20.76 |
| Glycine | 1.77 |
| Proline | 10.24 |
| Serine | 5.40 |
| Tyrosine | 5.40 |
| Total NEAA | 59.78 |
|  |  |
| Total | 100 |
