# Supplementary material for: An Elemental Diet Enriched in Amino Acids Alters the Gut Microbial Community and Prevents Colonic Mucus Degradation in Mice with Colitis
Source: mSystems. 2022 Dec 5;7(6):e00883-22. doi: 10.1128/msystems.00883-22 (PMC9765100; doi:10.1128/msystems.00883-22)
Supplement: TABLE S2 [file msystems.00883-22-s0003.docx]

**Table S2.**

| Gene | Forward Reverse | Forward Reverse |
| --- | --- | --- |
| *TNF-α* | GCTCTGTGAAGGGAATGGGTGTT | GCTCTGTGAAGGGAATGGGTGTT |
| *IL-1β* | GAAGAAGAGCCCATCCTCTG | GTTCATCTCGGAGCCTGTAG |
| *IL-6* | TAGTCCTTCCTACCCCAATTTCC | TTGGTCCTTAGCCACTCCTTC |
| *IL-12* | CACGATGAACCTGGAAACTC | CAAAGATGCTGGACTGGAAG |
| *IL-23* | TTCCCTGGGCATCAACATAC | ACCAAGGGCAGAAAGAAGAG |
| *IL-17A* | CAGACTACCTCAACCGTTCCAC | CAGCTTTCCCTCCGCATT |
| *IL-10* | GCACTACCAAAGCCACAAGG | GTAAGAGCAGGCAGCATAGC |
| *TGF-β* | AAGGACCTGGGTTGGAAGTG | CCGGGTTGTGTTGGTTGTAG |
| *IFN-γ* | TTTAACAGCAGGCCAGACAG | TCATTCGGGTGTAGTCACAG |
| *RegⅢγ* | CAAGAAGCTGAGCGAGTGTC | GTCCACGTCAGCAATCATCC |
| *ZO-1* | TTCACGCAGTTACGAGCAA | TTGGTGTTTGAAGGCAGAGC |
| *Occludin* | GGGCATTGCTCATCCTGAAG | GCCTGTAAGGAGGTGGACTT |
| *Muc2* | GCTGACGAGTGGTTGGTGAATG | GATGAGGTGGCAGACAGGAGAC |
| *Muc3* | AAAGATTACCTCCCATCTCC | TAAAACTAAGCATGCCCTTG |
| *Claudin-1* | AAAGCACCGGGCAGATACAG | CCCAGCAGGATGCCAATTAC |
| *Claudin-2* | AAGGACGGCTCCGTTT | TGGCAGACCTCTCAGTAGAA |
| *Claudin-3* | CGTACCGTCACCACTACCAG | CTGTGTGTCGTCTGTCACCA |
| *ICAM-1* | TCACATGGGTCGAGGGTTTC | ACCACTGCCAGTCCACATAG |
| *Relmβ* | TCATCACCAAGGCCATCGAG | TGTTGCTGTCGCCCTTATCC |
| *Foxp3* | ACTCTGCCTTCAGACGAGAC | GGTTGGGCATTGGGTTCTTG |
| *β-actin* | GGCTGTATTCCCCTCCATCG | CCAGTTGGTAACAATGCCATGT |
