# Supplementary material for: An Elemental Diet Enriched in Amino Acids Alters the Gut Microbial Community and Prevents Colonic Mucus Degradation in Mice with Colitis
Source: mSystems. 2022 Dec 5;7(6):e00883-22. doi: 10.1128/msystems.00883-22 (PMC9765100; doi:10.1128/msystems.00883-22)
Supplement: TEXT S1 [file msystems.00883-22-s0001.docx]

**16S rRNA high-throughput sequencing and bioinformatics analysis**

We amplified the V3–V4 hypervariable regions of bacterial 16S rRNA using primers 338F and 806R. The sequencing was conducted according to the standard protocols (Majorbio Bio-Pharm Technology Co. Ltd, Shanghai, China).

Bioinformatics analysis was carried out using the I-sanger platform (www.i-sanger.com). The classification of each 16S rRNA gene sequence was analyzed by the RDP Classifier algorithm (http://rdp.cme.msu.edu/) using a 70% confidence threshold against the Silva (SSU127) 16S rRNA database. After that, principal coordinate analysis (PCoA) was carried out using the R package, and the statistical analysis was performed based on PC1 and PC2 values with bray-Curtis distance.
