# Supplementary material for: An Elemental Diet Enriched in Amino Acids Alters the Gut Microbial Community and Prevents Colonic Mucus Degradation in Mice with Colitis
Source: mSystems. 2022 Dec 5;7(6):e00883-22. doi: 10.1128/msystems.00883-22 (PMC9765100; doi:10.1128/msystems.00883-22)
Supplement: TABLE S3 [file msystems.00883-22-s0004.docx]

**Table S3. Primers for the measurement of bacterial 16S rRNA genes by qPCR.**

| Gene | Forward | Reverse |
| --- | --- | --- |
| *A. muciniphila* | CAGCACGTGAAGGTGGGGAC | CCTTGCGGTTGGCTTCAGAT |
| *Bacteroides* | GGTTCTGAGAGGAGGTCCC | CTGCCTCCCGTAGGAGT |
| *B. thetaiotaomicron* | TACTCGCCTCTTTGCAACCCTACC | GGCCCCAGATCCGAACAACAC |
| *B. fragilis* | ATAGCCTTTCGAAAGRAAGAT CCAGTATCAACTGCAATTTTA | ATAGCCTTTCGAAAGRAAGAT CCAGTATCAACTGCAATTTTA |
| *B. caccae* | GGCGCATGACATTGGAGGTTT | AATACGCCGCATCGCTTTTTC |
| *B. vulgatus* | CGATTGGTCTGGCACGTATG | ACTTCATTGTCACGCACATTCAT |
| *B. uniformis* | GCTACCGGGAGATACTGGATTGG | TGCGGCGGCCTTTGAAC |
| *B. ovatus* | GTGAAGGTGCCATCGGAGGAC | GGACGCTTTGGCCACTATTTCA |
| Eubacteria | GGCTGTATTCCCCTCCATCG | CCAGTTGGTAACAATGCCATGT |
